# Supplementary material for: Targeted changes in blood lipids improves fibrosis in renal allografts
Source: Lipids Health Dis. 2023 Dec 4;22:215. doi: 10.1186/s12944-023-01978-x (PMC10694909; doi:10.1186/s12944-023-01978-x)
Supplement: Supplementary file 1 — Additional file 1. [file 12944_2023_1978_MOESM1_ESM.pdf]

files
Nov 21, 2023
3431 words / 19938 characters

GYNGH_26_7_Plagiarism_Check

Sources Overview

9%
OVERALL SIMILARITY

	Source	Type	Similarity
1	www.science.gov	INTERNET	1%
2	www.science.org	INTERNET	<1%
3	www.mdpi.com	INTERNET	<1%
4	&NA;, . "Poster Sessions : Kidney", Transplantation Journal, 2014.	CROSSREF	<1%
5	Shuai Yu, Hongmei Li, Taihua Cui, Mingxun Cui et al. "Onion (Allium cepa L.) peel extract effects on 3T3-L1 adipocytes and high-fat diet-...	CROSSREF	<1%
6	nanoscalereslett.springeropen.com	INTERNET	<1%
7	Hong, J.E.. "Solid-phase microextraction with on-fiber derivatization for the determination of hydroxy-polychlorinated biphenyl compo...	CROSSREF	<1%
8	Yu-Lin Wu, Si-Han Huang, Chun-Mei He, Bo Qiu, Jing-Jing Liu, Jia Li, Ying Lin, Sheng-Lu Yu, Hong-Feng Wang, Gui-Fang Zhang. "Dendro...	CROSSREF	<1%
9	docksci.com	INTERNET	<1%
10	"American Transplant Congress 2007 Executive and Program Planning Committees and Abstract Review Committees", American Jour...	CROSSREF	<1%
11	Preprint source<br>Li Li, YiJia Gong, Ke Xu, WeiHong Chen et al. "ZBTB28 induce autophagy via regulating FIP200 and Bcl-XL to facilitate apoptosis of cer...	CROSSREF POSTED CONTENT	<1%
12	www.frontiersin.org	INTERNET	<1%
13	link.springer.com	INTERNET	<1%
14	rep.bioscientifica.com	INTERNET	<1%
15	A. L. Croci Chiocchini, C. Sportoletti, G. Comai, S. Brocchi et al. "Correlation Between Renal Cortical Stiffness and Histological Determin...	CROSSREF	<1%
16	K.W. Lee. "The role of omega-3 fatty acids in the secondary prevention of cardiovascular disease", QJM, 07/01/2003	CROSSREF	<1%
17	"Orals", Transplant International, 2015.	CROSSREF	<1%

18 Benjamin A. Adam, Zeljko Kikic, Siegfried Wagner, Yassine Bouatou et al. "Intragraft Gene Expression in Native Kidney BK Virus Nephro... <1%
CROSSREF

19 Ge Chenxu, Zhong Shaoyu, Lai Lili, Xianling Dai, Qin Kuang, Li Qiang, Hu Linfeng, Lou Deshuai, Tan Jun, Xu Minxuan. "Betacyanins atte... <1%
CROSSREF

20 Ning Li, Ke Chen, Jinrong Bai, Zangjia Geng et al. "Tibetan medicine Duoxuekang ameliorates hypobaric hypoxia-induced brain injury in ... <1%
CROSSREF

21 R.E. Newman, L.H. Storlien, W.L. Bryden†, A.C. Kirby, J.A. Downing. " Dietary -3 and -6 Fatty Acids Alter Avian Pituitary Sensitivity ", Nut... <1%
CROSSREF

22 Stefano Barabino, Jutta Horwath-Winter, Elisabeth M. Messmer, Maurizio Rolando, Pasquale Aragona, Shigeru Kinoshita. "The role of ... <1%
CROSSREF

23 Yuejin Chen, Donald B. Hoover. "Autoradiographic localization of NK1 and NK3 tachykinin receptors in rat kidney", Peptides, 1995 <1%
CROSSREF

24 rupress.org <1%
INTERNET

25 test2.scicrunch.org <1%
INTERNET

Excluded search repositories:

- None

Excluded from document:

- Bibliography
- Quotes

Excluded sources:

- None

Excluded preprints

- None

Abstract

Background

Chronic interstitial fibrosis is the primary barrier against the long-term survival of transplanted kidneys. Extending the lifespan of allografts is vital for ensuring the long-term health of patients undergoing kidney transplants. However, few targets and their clinical applications have been identified. Moreover, whether dyslipidemia facilitates fibrosis in renal allograft remains unclear.

Methods

Blood samples were collected from patients who underwent kidney transplantation. Correlation analyses were conducted between the Banff score and body mass index, and serum levels of triacylglycerol, total cholesterol, low-density lipoprotein cholesterol, and high-density lipoprotein cholesterol. A rat model of renal transplantation was treated with the lipid-lowering drug, fenofibrate, and kidney fibrosis levels were determined by histochemical staining. Targeted metabolomic detection was conducted in blood samples from patients who underwent kidney transplantation and were divided into fibrotic and non-fibrotic groups. Rats undergoing renal transplantation were fed either an n-3 or n-6 polyunsaturated fatty acid (PUFA)-enriched diet. Immunohistochemical and Masson's trichrome staining were used to determine the degree of fibrosis.

Results

Hyperlipidemia was associated with fibrosis development. Treatment with fenofibrate contributed to improve fibrosis in a rat model of renal transplantation. Moreover, n-3 polyunsaturated fatty acids (PUFAs) from fibrotic group showed significant downregulation compared to patients without fibrotic renal allografts, and n-3 PUFAs-enriched diet contributed to delayed fibrosis in a rat model of renal transplantation.

Conclusions

This study suggests that hyperlipidemia facilitates fibrosis of renal allografts. Importantly, a new therapeutic approach was provided that may delay chronic interstitial fibrosis in transplanted kidneys by augmenting the n-3 PUFA content in the diet.

Background

When alternative therapies have been exhausted, kidney transplantation is the sole solution for end-stage renal disease. Owing to a dearth of available donor kidneys, extending the lifespan of transplanted kidneys has become necessary. Nevertheless, sustaining renal allografts over the long term presents considerable difficulties. Chronic allograft nephropathy was redefined as interstitial fibrosis and tubular atrophy[1], and represents a major factor affecting the long-term survival of renal allografts[2]. Renal allograft fibrosis is a multifactorial disease characterized by inflammation, T-cell-mediated rejection, immunosuppressive drug toxicity, and the epithelial-mesenchymal transition, all of which are closely associated with the development of fibrosis[3-5]. However, few targets and clinical applications to this end have been discovered. Moreover, whether dyslipidemia facilitates fibrosis in renal allograft remains unclear.

Polyunsaturated fatty acid (PUFA) are fatty acids with more than two unsaturated bonds. Linoleic acid (LA) and α-linolenic acid (α-LA) are essential fatty acids. They function as precursors for the biosynthesis of PUFAs by adding unsaturated bonds and elongating the fatty acid chain. PUFAs are classified based on the distance between the unsaturated bonds and the first methyl group. PUFAs are defined as n-3 PUFAs when the unsaturated bond is situated at the third carbon atom and as n-6 PUFAs when the unsaturated bond is situated at the sixth carbon atom[6]. PUFAs participate in several cellular processes and can affect disease progression[7]. PUFAs in the blood of patients with

liver cirrhosis were decreased compared to that in the control group, and supplementary essential fatty acids and long-chain PUFAs contributed to improvement of the cirrhosis[8]. In addition, n-3 PUFAs are risk predictors of heart failure[9]. A recent study demonstrated a positive correlation between elevated n-3 PUFA levels in the bloodstream and improved survival rates for renal allografts[10]. However, whether the addition of n-3 PUFAs improves fibrosis in renal allografts remains unclear.

One study revealed that hyperlipidemia significantly contributes to the development of fibrotic lesions in renal allografts. Treatment with lipid-lowing drug fenofibrate contributed towards improving fibrosis in a model of rat kidney transplantation. Analysis of blood sample collected from renal transplant recipients showed that the reduction of n-3 PUFAs was associated with fibrosis progression in renal allografts. An n-3 PUFA enriched diet attenuated fibrosis in a rat renal transplant model. Based on the results from blood samples of renal transplant recipients and rat models of renal transplantation, a low fat and high-proportioned PUFA diet may be beneficial to the extended upkeep of renal allografts.

Materials and methods

Blood specimens

Patients were recruited from the First Hospital of Jilin University, China. All clinical records were obtained with the approval of the Ethics Committee. Clinical records were divided into three groups; 69 cases with ci0, 63 cases with ci1, and 19 cases with ci2/3. Targeted lipidomic detection used blood samples from seven patients with ci0, six patients with ci1, and one patient with ci2. There was a partial overlap in the above samples for different projects.

Rat kidney transplantation model

Sprague-Dawley rats (180-200g) were used as donors and receptors. Prior to operation, anesthesia was administered to the mice using isoflurane. For the donor, heparinized saline (at a concentration of 125 U/mL) was injected, and the blood supply was halted using styptic clips. The left renal vein was removed before perfusion. The left renal vein, ureter, and artery were ligated after perfusion was complete. Heparinized saline (0~4°C) was used as a preserving fluid for the separated left kidney. For the recipient, the left kidney, renal artery, renal vein, and ureter were separated and cut off. The renal artery, vein, and ureter were then anastomosed between the donor and recipient before the blood supply was restored. Bladder anastomoses were created to avoid ureteral obstruction, which may lead to unsuccessful ureter anastomosis. For postoperative management, 5 mL saline and ketoprofen (5 mg/kg) were injected into the abdominal cavity before the abdomen was closed. After renal transplantation, penicillin was administered for 3 days and cyclosporin A for 1 week. The animals were included in the study if they underwent successful renal transplantation, defined by improved blood supply of renal veins and arteries.

Immunofluorescence staining

The sections were dewaxed and washed with water conventionally. Thermal antigen retrieval with citrate for 5 min at 95°C was performed. 5% BSA was used to block the sections for 30 min before being incubated with the primary antibodies, including antibodies against acyl-CoA oxidase 1 (ACOX1) (DF12046, Affinity Biosciences, Ohio, USA) and α-smooth muscle actin (α-SMA) (ab7817, Abcam, Cambridge, UK), in PBS containing 1% BSA, and the sections were incubated overnight at 4°C. The sections were washed thrice before being incubated with the different fluorescent dye–conjugated secondary antibodies for 1 h. After washing the sections three times with PBS, Lotus Tetragonolobus Lectin (LTL) (FL-1321-2, Vector, California, US) was used for

incubation for 30 min. The sections were then washed thrice before the cell nuclei were stained with DAPI for 10 min. After washing them again thrice, the sections were sealed with antifade mounting medium (P0128, Beyotime, Shanghai, China). The sections with stained tissues were mounted onto a confocal microscope and images were captured.

Hematoxylin & eosin.

Sections were dewaxed twice with xylene for 30 min, then dipped into a concentration gradient of ethyl alcohol for 5 min. They were then washed with distilled water and subjected to 5 min of hematoxylin staining, differentiated with acid alcohol, blued with ammonia water, and subjected to a 10 min eosin staining procedure. The sections were dehydrated with a gradient of 70%, 80%, 95% I, 95% II, 95% III, 100% I, and 100% II ethyl alcohol. The samples were immersed twice in xylene for a duration of 10 min before they were sealed with neutral resins.

Masson

The sections underwent a range of procedures, including dewaxing, staining with hematoxylin and ponceau-acid fuchsin solution, washing with 2% glacial acetic acid, differentiation with 1% phosphomolybdic acid, staining with aniline blue, dehydrating, and sealed with neutral resin.

Targeted lipidomics

A standard solution was prepared. Fatty acid calibrators at concentrations of 10–40000 ng/mL were prepared by mixing the stock solutions of individual fatty acids with a fatty acid-free matrix. The samples were resuspended and homogenized in liquid nitrogen with 300 μL of a mixture of isopropanol and acetonitrile (1:1) containing internal standards, which were mixed. The samples were subsequently underwent centrifugation at 12,000g for a duration of 10 min. The 2 μL of supernatant was extracted and injected into the LC-MS/MS system. The fatty acids were quantified

using a UHPLC-MS/MS system. The concentration series of the standard solutions were detected using LC-MS. The ratio of standard to internal standard concentrations was determined using the x-axis, whereas the y-axis, denoting the peak area of the standard to internal standard, was designated as the ordinate to assess the linearity of the standard solution. Each metabolite was allowed to reach a correlation coefficient (r) > 0.99. The limit of quantification was determined using the signal-to-noise ratio method.

Immunohistochemistry (IHC)

The sections were dewaxed and washed with water conventionally. Thermal antigen retrieval with citrate for 5 min at 95°C was performed. An ultra-sensitive ™ SP IHC kit procured from MXB biotechnologies (KIT-7710, Fujian, China) was used for IHC detection. Tissues then underwent the following treatment steps: incubation with Reagent I for 15 min, washed thrice with PBS for 5 min, and incubation with Reagent II for 15 min. The tissues were then incubated with dilute specific antibodies, including Fibrillin 1 1:200 (AF0429, Affinity Biosciences, Ohio, USA), MMP7 1:4000 (AF0218, Affinity Biosciences, Ohio, USA), and collagen IV 1:400 (AF0510, Affinity Biosciences, Ohio, USA), at 4 °C overnight. The next day, the tissues were subjected to three washes with PBS and were then incubated with Reagent III. Subsequently, the tissues were washed again with PBS, incubated with Reagent IV, and then washed three more times with PBS. Subsequently, 3,3-diaminobenzidine (DAB) staining was performed until tissues were stained with brown or red, then terminated with water. In this step, one antibody performed same dyeing time. Cell nuclei were visualized by staining the sections with hematoxylin. To retain the sample, the sections were dehydrated and preserved using neutral resin.

Total cholesterol (TC) and triacylglycerol (TG) detection in rat blood

Serum was collected from rats with renal transplantation. TC (A111-1-1) and TG (A110-1-1) detection kits were obtained from Nanjing Jiancheng Bioengineering Institute, located in Nanjing, China. Serum (2.5μL) or standard substance (TG 2.39mmol/L, TC 6.15mmol/L) was maximixed with working solution in 96 pore plate and subsequently incubated at 37°C for 10 min. Absorbance was measured at a wavelength of 500 nm. TC and TG concentrations were counted using the subsequent equations: TG (mmol/L) = (A_{sample}-A_{blank}/$A_{standard}$-A_{blank})x 2.39 mmol/L; TC (mmol/L) = (A_{sample}-A_{blank}/$A_{standard}$-A_{blank}) x 6.15 mmol/L.

Statistical analysis

GraphPad Prism 9, developed by GraphPad Software in California (USA), was utilized to perform statistical analyses in this study. The data have been provided as means ± standard errors of the means. Statistical significance was determined using Student's *t*-tests, where *$P < 0.05$, **$P < 0.01$, ***$P < 0.001$, and ****$P < 0.0001$ signify statistical significance.

Results

Disordered lipid metabolism in blood is associated with interstitial fibrosis in renal allografts

Blood samples were collected during various post-transplantation periods from 151 patients who underwent kidney transplantation. To confirm whether obesity was associated with fibrosis in transplanted kidneys, the relationship between fibrosis and body mass index (BMI) was analyzed; level of fibrosis was measured by ci-scores ranging from 0 to 3 to determine the progress of fibrosis according to the Banff score. BMI did not correlate with the ci-score (Fig. 1A). Early causes or acute reactions, such as ischemia reperfusion and acute rejection, also result in injury-induced fibrosis. Thus, to exclude acute injury-induced fibrosis in transplanted kidneys, cases that had undergone transplantation more than 5 years prior were analyzed. The percentage of overweight patients

(BMI>25) displayed an increasing trend along with the progression of fibrosis (Fig. 1B). The results revealed that the amount of TG in patients with ci-scores of 2 and 3 was significantly higher than that in patients with ci-scores of 0 or 1 (Fig. 1C). Further analysis revealed that cases with percentages of high TG (>1.75 mmol/L) accounted for 52.00% of ci0 cases, 67.57% of ci1 cases, and 85.71% of ci 2/3 cases (Fig. 1D). In addition, TC levels were higher in ci 2/3 patients than in ci 0 and ci 1 patients (Fig. 1E). Cases with percentage of high TC (>5.2 mmol/L) accounted for 64.29% which was far higher than ci 0 and ci 1 cases (Fig. 1F). A correlation analyses of BMI, TG, TC, low-density lipoprotein cholesterol (LDL-C), and high-density lipoprotein cholesterol (HDL-C) with several other indices of the Banff score were further conducted, including total interstitial inflammation (ti), tubulitis (t), glomerulitis (g), C4d deposition, vascular fibrous intimal thickening (cv), and arteriolar hyaline thickening (ah), in cases where transplantation was performed more than 5 years prior. The results showed that the proportion of patients with high TG and LDL-C levels progressively increased alongside cv and ah progression (Table 1). These findings indicate that abnormal lipid levels in blood are correlated with fibrotic lesions.

Fenofibrate attenuates fibrosis in a rat model of renal transplantation

Fenofibrate activates peroxisome proliferator-activated receptor alpha (PPARα), which has a critical function in promoting fatty acid oxidation[11]. This drug is used for the treatment of hypertriglyceridemia and mixed dyslipidemia[12]. As renal transplant patients with serious fibrosis display increased blood TG and TC levels, rat models of renal transplantation were treated with fenofibrate for four weeks (Fig. 2A). Fenofibrate treatment increased the expression of CD36 and ACOX1 in liver which were responsible for the uptake and oxidation of lipid (Fig. 2B, C). TG and TC levels in blood were decreased after fenofibrate treatment (Fig. 2D), indicating that fenofibrate

treatment produced the expected effects. Following treatment with fenofibrate, transplanted kidneys displayed a ruddier color and softer grains than those in the untreated group (Fig. 2E). Immunofluorescence (IF) detection showed that fenofibrate also upregulated ACOX1 expression in kidney, and decreased α-SMA expression (Fig. 2F), indicating that it may decrease lipid level in renal allograft. Hematoxylin-eosin staining showed that fenofibrate treatment improved kidney injury in the rat models of renal transplantation (Fig. 2G), while Masson's and Sirius red staining showed that fenofibrate treatment inhibited the development of fibrosis (Fig2. G, H). Taken together, the lipid-lowering drug, fenofibrate, delayed the progression of fibrosis in renal allografts.

The n-3 PUFA level is decreased in serum from renal transplantation recipients with fibrosis

To ascertain the association between the serum lipid composition and fibrosis progression, blood samples were collected from patients who underwent kidney transplantation more than five years prior, including seven patients with ci-score 0 (Group A), six cases with ci-score 1, and one case with ci-score 2 (Group B) (Fig. 3A). There was no statistically significant difference in total TG levels between the two groups (Fig. 3B). Blood samples were also analyzed using targeted metabolomics to determine the free fatty acid content. The results showed that saturated fatty acids (SFAs) were unchanged between groups A and B (Fig. 3 C). However, the majority of PUFAs decreased in the blood of patients with allograft fibrosis (Fig. 3D, E). It is noteworthy that n-3 PUFAs showed more significant decrease than n-6 PUFAs, especially the essential fatty acid α-LA. In summary, serum obtained from fibrotic renal transplantation recipients showed decreased PUFA levels compared to that of non-fibrotic patients, proposing that n-3 PUFAs may affect fibrosis progression in renal allografts.

An n-3 PUFA enriched diet contributes to improved fibrosis in a rat renal transplant model

Targeted metabolomics results showed that LA was not altered in either group; nevertheless, α-LA was significantly decreased in Group B (Fig. 3D, E). This suggests that although both n-3 and n-6 PUFAs are necessary to prevent fibrosis in renal allografts, a lack of n-3 PUFAs may play a more crucial role in facilitating fibrosis. To verify whether the addition of n-3 PUFAs alleviated fibrosis progression in renal allografts in vivo, a rat renal transplantation model was established and the rats were supplied with soybean oil feed (SbOF), a type of edible oil, or linseed oil feed (LOF) (Fig. 4A). The total lipid content in both feeds was 7%. SFAs and monounsaturated fatty acids were present in similar proportions. The biggest difference between the feeds was that soybean oil includes more n-6 PUFAs, whereas linseed oil has a higher amount of n-3 PUFAs (Fig. 4B). Compared with normal kidneys (right), transplanted kidneys (left) showed a lower volume (Fig. 4C). In addition, transplanted kidneys in rats fed with SbOF were smaller in volume, whiter in color, and tougher in grains than those treated with LOF (Fig. 4C). The n-3 PUFA diet effectively alleviated the development of fibrosis in renal allografts (Fig. 4D, E) and did not produce observable lesions in other organs (Fig. 4F). The IHC analyses revealed that the n-3 PUFA diet downregulated the expression of fibrosis-related proteins, including collagen IV (Col 4), matrix metalloproteinase 7 (MMP7), and fibrillin 1 (FBN1) (Fig. 4G, H). In addition, LOF did not affect the expression of ACOX1 and CD36 in liver (Fig. 4I, J) or the TG and TC levels in blood (Fig. 4H), indicating that n-3 PUFA diet improved fibrosis in renal allograft and the desired effects were not dependent on reducing blood lipid levels. These findings suggest that augmenting the n-3 PUFA content in the diet may delay chronic interstitial fibrosis in renal allografts.

Discussion

Chronic interstitial fibrosis is a major challenge in patients undergoing kidney transplantation.

Preventing the progression of fibrosis and prolonging the survival time of renal allografts are urgent issues. Although multiple underlying mechanisms for renal fibrosis have been elucidated[5], few therapeutic targets have been developed into applications suitable for use in clinical practice.

Obesity is a critical factor that accelerates liver fibrosis in the development of non-alcoholic fatty liver disease[13]. However, the contribution of blood fat to the progression of renal allograft fibrosis remains unclear. The results showed that hyperlipidemia may contribute to the progression of fibrosis in renal allografts. Patients who undergo renal transplantation are usually instructed to control their diet, and while fatty acid oxidation is the primary energy supplement for the kidneys, fibrotic renal tissue cannot oxidize enough fatty acids, resulting in lipid accumulation in the kidneys and blood. Fibrotic renal allografts, therefore, may induce hyperlipidemia.

Fenofibrate has been extensively used clinically to lower blood lipid levels[14]; however, it also functions at the cellular level. While the results showed that lowering blood lipid with fenofibrate improved fibrosis in a rat model of renal transplantation, the possible effect of the drug on cells cannot be excluded. For example, a recent study has shown that fenofibrate affects the vitality, function, and phenotype of proximal tubular cells[15], macrophages[16], and T cells[17], which are critical factors influencing fibrosis in renal allograft fibrosis. In addition, a previous study reported that fenofibrate improved fibrosis by inhibiting oxidative stress in renal allografts[18]. Unquestionably, fenofibrate is a potential agent for delaying fibrosis in renal allografts though its molecular mechanism requires further elucidation.

In addition to energy supplement, fatty acids such as arachidonic acid, play a vital role in signal transduction[19]. The results showed that serum PUFA levels were decreased in fibrotic renal transplant patients; however, the causality as to why PUFAs decreased remains unclear. Fibrotic

renal tissues consume excess PUFAs, giving rise to implications that n-3 PUFAs being consumed may be leading to rise in some stress status. Consistent with this understanding, n-3 PUFA supplementation provenly improves oxidative stress in mice with chronic kidney disease[20], and patients with intestinal failure[21]. In addition, n-3 PUFA supplementation inhibits endoplasmic reticulum stress[22]. Both oxidative and endoplasmic reticulum stress play critical roles in the fibrosis of renal allografts [23, 24]. Thus, a diet supplemented with n-3 PUFAs may improve fibrosis in renal allografts by suppressing stress.

Strengths and limitations

This study effectively revealed the association between dyslipidemia and fibrosis progression in renal allografts. Changes in blood lipid levels following treatment with lipid-lowering drug fenofibrate and an n-3 PUFAs enriched diet are effective approaches towards maintaining the long-term survival of renal allografts. Fenofibrate is commonly used in clinical medicine and n-3 PUFAs are essential fatty acids for humans, indicating that both these approaches had the potential of clinical transformation. Several limitations also exist in this study. First, abnormal lipid metabolism may affect the function of immune cells and the transformation of epithelial cells. Future research should be focused on the mechanistic area of study. Second, LOF was a complicated component, it will be constructive to specify which n-3 PUFAs play the critical role in suppressing fibrosis in renal allografts.

Conclusion

Taken together, these findings suggest that hyperlipidemia is a risk factor for the advancement of fibrosis in renal allografts. The lipid-lowering drug fenofibrate is a potential therapeutic agent for the treatment of fibrosis. These findings support a new therapeutic approach that may delay chronic

interstitial fibrosis in renal allografts by augmenting the dietary n-3 PUFA content. This study indicated that patients who underwent renal transplantation should follow a dietary habit, a low fat but high-proportioned PUFAs diet, which may contribute in delaying the survival time of a renal allograft.
